# Transcriptional and apoptotic responses of THP-1 cells to challenge with toxigenic, and non-toxigenic *Bacillus anthracis*

**DOI:** 10.1186/1471-2172-9-67

**Published:** 2008-11-13

**Authors:** Christopher Bradburne, Myung-Chul Chung, Qin Zong, Karen Schlauch, Derong Liu, Taissia Popova, Anna Popova, Charles Bailey, Dan Soppet, Serguei Popov

**Affiliations:** 1 Center for Bio/Molecular Science and Engineering Code 6900, US Naval Research Laboratory, Washington DC, USA; 2 National Center for Biodefense and Infectious Disease, George Mason University, Manassas, VA, USA; 3 Avalon Pharmaceuticals, Germantown, MD, USA; 4 Department of Biochemistry and Molecular Biology, University of Nevada Reno, Reno, Nevada, USA; 5 College of Arts and Sciences, University of Virginia, Charlottesville, VA, USA

## Abstract

**Background:**

*Bacillus anthracis *secretes several virulence factors targeting different host organs and cell types during inhalational anthrax infection. The bacterial expression of a key virulence factor, lethal toxin (LeTx) is closely tied to another factor, edema toxin (EdTx). Both are transcribed on the same virulence plasmid (pXO1) and both have been the subject of much individual study. Their combined effect during virulent anthrax likely modulates both the global transcriptional and the phenotypic response of macrophages and phagocytes. In fact, responses brought about by the toxins may be different than each of their individual effects.

**Results:**

Here we report the transcriptional and apoptotic responses of the macrophage-like phagocytic cell line THP-1 exposed to *B. anthracis *Sterne (pXO1^+^) spores, and *B. anthracis *Δ Sterne (pXO1^-^) spores. These cells are resistant to LeTx-induced cytolysis, a phenotype seen in macrophages from several mouse strains which are sensitive to toxigenic anthrax infection. Our results indicate that the pXO1-containing strain induces higher pro-inflammatory transcriptional responses during the first 4 hours of interaction with bacterium, evident in the upregulation of several genes relevant to Nf-κB, phosphatases, prostaglandins, and TNF-α, along with decreases in expression levels of genes for mitochondrial components. Both bacterial strains induce apoptosis, but in the toxigenic strain-challenged cells, apoptosis is delayed.

**Conclusion:**

This delay in apoptosis occurs despite the much higher level of TNF-α secretion induced by the toxigenic-strain challenge. Interestingly, CFLAR, an important apoptotic inhibitor which blocks apoptosis induced by large amounts of extracellular TNF-α, is upregulated significantly during toxigenic-strain infection, but not at all during non-toxigenic-strain infection, indicating that it may play a role in blocking or delaying TNF-α-mediated apoptosis. The suppression of apoptosis by the toxigenic anthrax strain is consistent with the notion that apoptosis itself may represent a protective host cell response.

## Background

Highly pathogenic strains of *Bacillus anthracis *contain two plasmids, XO1 and XO2 encoding major virulence factors of this Gram-positive bacillus. Lethal toxin (LeTx) and edema toxin (EdTx) genes reside on the pXO1, while the anti-phagocytic capsule is encoded by pXO2. LeTx is necessary for pathogenicity, as deletion of its gene renders the microbe avirulent, while EdTx-knockout strains are only partially attenuated [1]. Capsule substantially contributes to the virulence of the microbe but unencapsulated strains, such as Sterne (34F2), are still capable of causing death in experimental animals [1,2] Therefore, the pXO1^+^, pXO2^- ^Sterne strain serves as a convenient experimental toxigenic model of highly virulent strains.

During inhalational exposure to *B. anthracis*, the spores may enter alveoli where they become deposited on mucosal surfaces. Within hours of the initial interactions with the host, the spores can be engulfed by phagocytes, such as monocyte-derived macrophages or dendritic cells [3,4]. Both LeTx and EdTx are expressed upon germination within the macrophage phagosome [3] and seem to play important roles in suppressing the bactericidal innate immune mechanisms of the epithelium and the intra-phagocytic environment [3,5-9]. In a currently accepted model of anthrax, some of the phagocytosed spores *en route *to the mediastinal lymph nodes survive and multiply within the phagolysosome, kill the cell, and become released into the lymphatic system [5]. In the following process of hemorrhagic lymph node destruction, the bacteria gain access to the bloodstream and quickly become systemic by spreading to the spleen and other internal organs[10,11]. According to this mechanism, lung phagocytes such as macrophages and dendritic cells are critically involved in the initiation of the disease, and their response to anthrax spores, among other factors, determines whether the exposure to aerosolized spores results in the infectious process [12].

Macrophages were the first cell type discovered to die after exposure to LeTx [13]. LeTx consists of a heptameric protective antigen (PA) noncovalently associated with lethal factor (LeF). LeTx is a zinc metalloprotease, which cleaves and thus inhibits mitogen-activated protein kinase kinase (MAPKK) family members *in vitro *and *in vivo*, resulting in defective host cell signaling [14-16], with broad implications for the host innate and adaptive immune responses [17]. However the death of macrophages after exposure to LeTx *in vitro *does not correlate with the cleavage of the MAPKK substrates by LeTx, and the macrophages sensitive to LeTx reside in the strains of mice tending to be resistant to the lethal effect of LeTx [2,18]. This paradoxical effect did not get a satisfactory mechanistic explanation till it was discovered that LeTx was able to induce the process of programmed, apoptotic death in a murine macrophage cell line RAW 264.7 [19]. It was suggested that for sensitive macrophages, and perhaps other cell types, undergoing apoptosis may serve as an *in vivo *sensor alerting the immune system and inducing the protective response [20]. Similar correlation between macrophage susceptibility to apoptosis and the outcome of infection in mice takes place, for example, in the case of *M. tuberculosis *[21-23]. Generally, pneumonic macrophages appear to exhibit a broad apoptotic response during less virulent *M. tuberculosis *infection, while infection with more virulent strains correlates with a near complete loss of the same apoptotic indicators [23].

Several studies have found that resistance of cells to LeTx may vary depending on the conditions of growth or stimulation. Human peripheral blood monocytes with the LeTx-resistant phenotype become sensitive to LeTx upon growth media deprivation leading to a cellular stress [6]. This finding was further elaborated, when it was shown that the stress factors *in vitro *sensitizing murine macrophages to apoptosis include a range of the bacterial Toll-like receptor (TLR) agonists of different nature, including the pore-forming hemolysins, peptidoglycan or endotoxin [24]. Human U-937, HL-60 and THP-1 cell lines of monocyte origin are resistant to LeTx but become sensitized upon phorbol myristate acetate (PMA)-induced differentiation into macrophages in culture [25], although relevance of this stimulation to the infection process *in vivo *is not clear. Additionally, PMA-induction may yield significant changes in the transcriptome which do not occur in *vivo*[26].

In contrast to the LeTx-exposed cells, human peripheral blood monocytes and monocyte-like undifferentiated THP-1 cells readily become apoptotic after exposure to *B. anthracis *spores [6]. These observations suggest that the process of infection may involve sensitization of cells to LeTx through the activity of unknown factors, such as the bacterial TLR agonists, similar to the effects of these agonists *in vitro*[24]. Another possibility is the existence of apoptotic factor(s) independent of LeTx or working in concert with it. In order to explore these hypotheses we decided to obtain insight into the transcriptional and apoptotic responses of THP-1 cells challenged with *B. anthracis *spores. To identify host signaling associated with the pathogenic activity of pXO1-encoded bacterial factors we compared the effects of the toxigenic (pXO1^+^, pXO2^-^) Sterne strain and the non-toxigenic (pXO1^-^, pXO2^-^) Δ Sterne strain. In experimental animals the toxigenic strain, even in the absence of capsule-encoding pXO2, is able to cause a lethal systemic infection, while a non-toxigenic strain is non-lethal and causes no clinical symptoms of disease. We therefore suggested that the use of the matched pair of isogenic strains would provide a means to distinguish between the pathogenic and the protective host responses.

We demonstrate that the presence of toxin plasmid XO1 in the challenge strain results in higher pro-inflammatory responses. Both *B. anthracis *strains induce apoptosis, albeit by different mechanisms. Our data supports a hypothesis that in the presence of pXO1, apoptosis may proceed through the mitochondria-dependent pathway, while in the non-toxigenic infection it seems likely to be activated mainly through an extrinsic TNF-α receptor-mediated caspase-8 pathway. The onset of apoptosis induced by a toxigenic strain is markedly delayed, and key apoptotic inhibitors are clearly induced, indicating that the suppression of early apoptosis in phagocytic cells may be beneficial for the virulent microbe. The fact that pXO1 is not required for the induction of apoptosis suggests that in addition to LeTx, *B. anthracis *possesses chromosomally-encoded pro-apoptotic factor(s).

## Results

### Growth characteristics indicate increased bacterial replication in the presence of THP-1 during early growth

**Figure 1:**
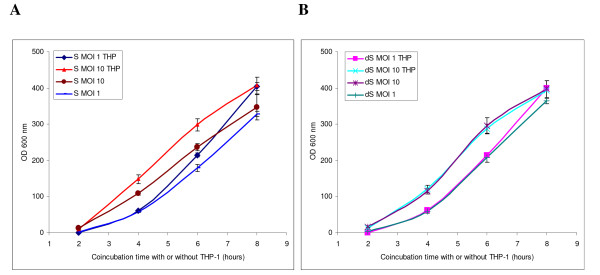
Incubation of A) *B. anthracis *Sterne, or B) *B. anthracis *Δ Sterne, in serum-free media with or without 1 × 10^6 ^THP-1 cells, and at multiplicities of infection (MOI) of either 1:1 or 1:10 of THP-1 to bacterium.

A *Bacillus anthracis *Δ Sterne strain was selected which was negative for the toxigenic XO1 plasmid, identical in genomic variable sequences checked, and which had identical growth characteristics to its parent Sterne strain (Additional files 1 and 2). THP-1 cells were challenged with spores of the Sterne strain and Δ Sterne strains, the bacterial growth monitored, and THP-1 mRNA was extracted at early time points to be used for microarray analysis. Figure 1 shows the bacterial growth represented by simple absorbance at 2 to 8 h post-coincubation with THP-1. In the non-toxigenic challenge, there is no statistically significant difference between challenge with or without THP-1 other than the MOI-dependent difference. This is in contrast to the Sterne-challenge, which indicates more bacterial replication in the presence of THP-1 at both MOIs.

### Principal component analysis indicates a suppressed response to toxigenic strains during early (2 h) infection

**Figure 2:**
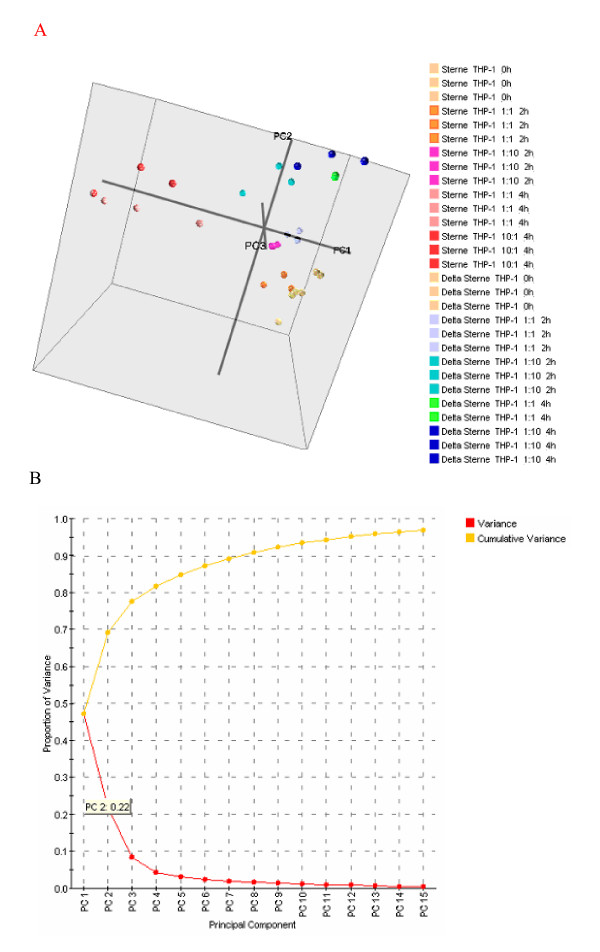
**A) Principle component analysis of microarrays run in this study confirms groupings of similar treatments, time points, and experimental replicates.** Each color corresponds to the same experimental treatment, while each point represents a separate chip. Axes labeled PC1, PC2, and PC3 and represent Principle Component 1, Principle Component 2, and Principle Component 3, respectively. B) Variance of principle components evaluated.

**Figure 3:**
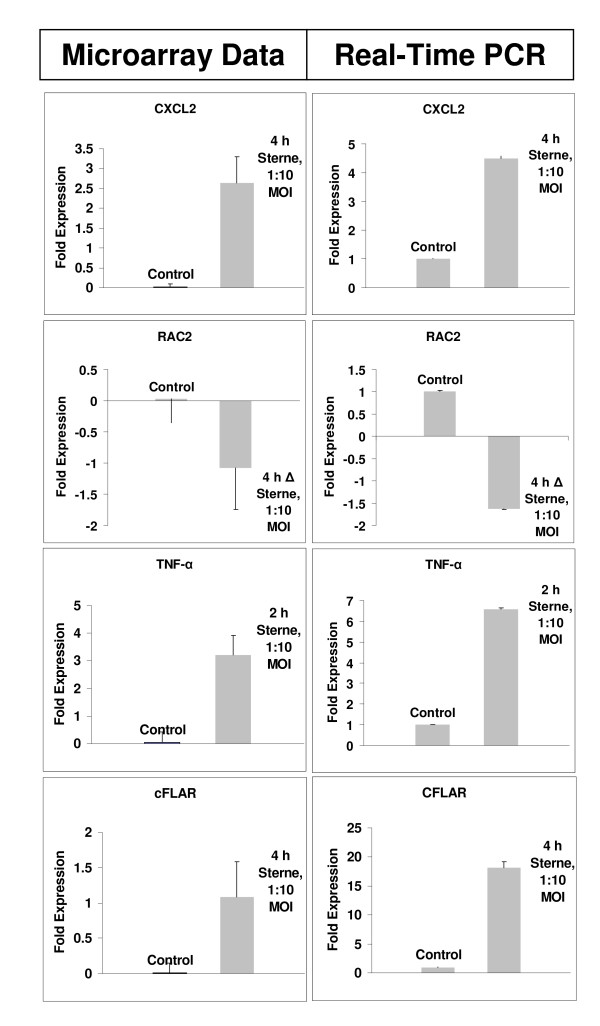
**Real-Time PCR of single gene treatments confirms trends in microarray expression levels.** Y-axis in microarray data (left) represents the fold change in gene expression relative to untreated control samples (labeled control) of THP-1 cells. The Y-axis in Real-Time PCR data (right) represents the fold gene expression in the THP-1 cells within the sample relative to the level of endogenous 18-S gene expression, and was calculated using the 2^-ΔΔCT ^method described by Livak and Schmittgen[69]. Labels within each panel indicate the time post- exposure, the challenge strain and the MOI.

THP-1 mRNA was collected at 0, 2, and 4 h time points, and the transcriptional response determined by microarrays. Principal component analysis of the microarray results shows time- and dose-dependent responses by THP-1 cells during challenge by both strains of *B. anthracis *(Fig. 2A). Variation attributed to each component is also shown (Fig 2B). Data points clustered in the PCA and confirmed that the principal components of the data set are the experimental variables such as MOI, post exposure time and the nature of the challenge strain. Therefore the mRNA expression levels reflect changes depending on the differences in treatment conditions rather than experimental artifacts. As expected, PCA also closely grouped the results with respect to chip replicates indicating their consistency, and allowed us to evaluate the overall dynamics of cell responses (Fig. 2). The results of the 2-hour challenges by toxigenic Sterne strain spores at both MOIs cluster in the 3-dimensional parameter space close to the control samples within the same quadrant of our PCA, in contrast to the cell responses to 2-hour challenges by Δ Sterne spores. This demonstrates that THP-1 cells respond to the non-toxigenic infection faster, compared to the toxigenic one, in agreement with the hypothesis that the initial general response of macrophages to the virulent spores is suppressed due to the presence of the LeTx-expressing plasmid [17]. At the 4-hour time point, there are notable and discernable differences between Sterne and Δ Sterne strain-challenged cells indicating a clear distinction in cellular responses between the strains with regard to the presence of the toxigenic plasmid. The strain-specific separation at higher MOIs and time points is so distinct that it may be useful in delineating toxigenic *vs*. non-toxigenic strains. Further confidence in our gene chip data was obtained using Real-Time PCR (Fig. 3), which demonstrates the results overall correlating to the microarray data within the experimental variations of both data sets.

### Gene ontology comparisons yield known and unknown differences

**Figure 4:**
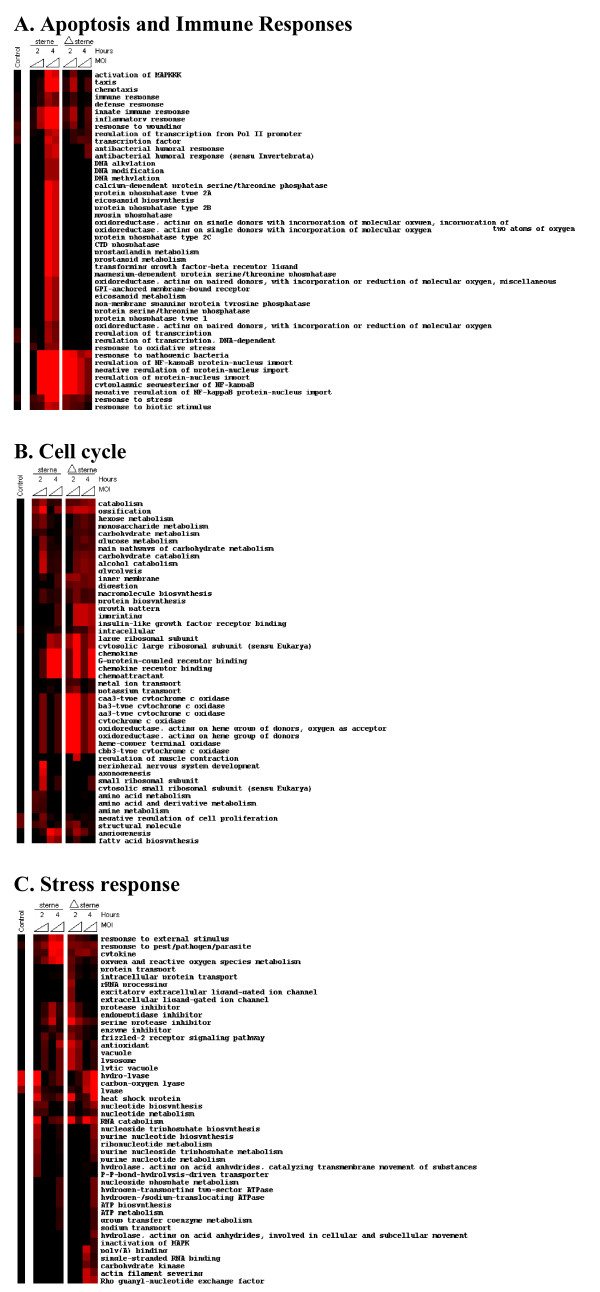
**Heatmaps of GO-type biological response modules show both similarities and differences in monocytic responses to Sterne and Δ Sterne *B. anthracis *strains.** MOIs correspond to a 1:1 at the low end of a triangle, and a 1:10 at the high end of each triangle. Color ranges represent no activation (black), low activation (faint red), and extensive activation (full red).

Fig. 4 shows clustered heatmaps of important biological processes, molecular functions, and cellular component modules. In general, our results for both strains appear consistent with the macrophage and innate immune activation programs described in response to other bacterial pathogens [27,28]. Activation of innate and other immune defenses and inflammatory responses are typically observed for both spore strains, but in general the activation is greater in the case of the more virulent Sterne strain spore-challenge model (Fig. 4a) in spite of the overall initial delay noticed in the 2-hour challenges. Several of the NF-κB-controlled programs are upregulated similarly between both strains, indicating that they are pXO1-independent, while the others could be considered as characteristics of virulence. The most striking differences seen between strain challenges in our clustering analyses consist of a series of modules that are activated at the 4-hour time points in the Sterne spore treatments, while no activation is seen in any of the Δ Sterne spore treatments. Among these modules, there are important signaling pathway processes, including at least 10 phosphatase modules, DNA modification modules, and transcriptional regulation modules. In addition, the pathways involved in eicosanoid and prostaglandin-type metabolism are similarly affected, which agrees with reports on drastic changes in prostaglandin levels during toxigenic-anthrax pathogenesis in mice [29]. Other cellular component functions encompassing catabolic and metabolic components do not show much variation between strains, similar to nucleotide/nucleic acid and ATP metabolism and catabolic processes (Fig 4b, c). Interestingly though, the cytochrome oxidase and oxidoreductase modules appear highly activated early in the avirulent Δ Sterne strain challenge, in comparison to the Sterne strain, marking the suppression of the transcriptional-level mitochondrial activation as an important virulence feature. Previous reports have indeed implicated LeTx in the dysfunction of mitochondria in murine macrophages and human peripheral blood monocytes [6,30].

### Anti-apoptotic and stress-responses are favored during toxigenic challenge, while cell cycle/differentiation responses are favored during non-toxigenic challenge

To further examine specific differences in host response to each strain, an Analysis of Variance (ANOVA) was performed and genes found to be significant in the cell survival, stress response, and apoptosis were compared between different strain challenges under identical conditions (Tables 1, 2, 3). The use of ANOVA here allowed us to evaluate the differential expression of a particular human gene while varying 3 conditions: time, MOI, and the presence or absence of pXO1 in the infecting *B. anthracis *strain. In our analysis, positive numbers indicate that the expression levels are higher in the non-toxigenic pXO1 (-) spore treatment, while negative numbers reflect a shift in expression level favoring the more virulent toxigenic pXO1 (+) spore treatment. A complete list of genes evaluated in the ANOVA can be found in Additional file 3 of the Additional Materials.

**Table 1:** Log ratios of genes of interest in ANOVA analysis* for apoptotic genes

**Accession**	**Name**	**Description**	**2 h 1:1 MOI**	**2 h 1:10 MOI**	**4 h 1:1 MOI**	**4 h 1:10 MOI**
NM_001196	BID	BH3 interacting domain death agonist	-0.0317	-0.0750	-0.6127	-0.5717
NM_015675	GADD45B	growth arrest and DNA-damage-inducible, beta	-0.0800	-0.5800	-2.0967	-2.0000
XM_046543	MKPX	mitogen-activated protein kinase phosphatase x	0.1183	0.5667	0.8563	0.8250
XM_010767	NCKAP1	NCK-associated protein 1	0.1750	0.2333	0.4737	0.2750
NM_006107	OA48-18	acid-inducible phosphoprotein	0.0433	0.0333	0.5737	0.7933
NM_004180	TANK	TRAF family member-associated NFKB activator	0.2383	-0.2633	-0.7193	-0.6650
NM_003879	CFLAR	CASP8 and FADD-like apoptosis regulator	-0.0583	-0.1283	-0.7720	-1.1250
XM_028204	NFKB1	nuclear factor of kappa light polypeptide gene enhancer in B-cells 1 (p105)	-0.1467	0.0283	-0.7677	-1.3933
XM_041847	TNF	tumor necrosis factor (TNF superfamily, member 2)	-0.1683	-0.7250	-2.4107	-1.3967
XM_012894	ZNF14	zinc finger protein 14 (KOX 6)	0.3217	0.2067	0.2593	0.1017
NM_014977	ACINUS	apoptotic chromatin condensation inducer in the nucleus	0.2867	0.5983	0.0723	-0.5367

*All adjusted P-values < 0.05. Ratios represent comparisons of expression levels of non-toxigenic vs. toxigenic-challenge. (A more comprehensive list of genes is shown in the additional materials, Additional file 3).

**Table 2:** Log ratios of genes of interest in ANOVA analysis* for cell cycle genes

**Accession**	**Name**	**Description**	**2 h 1:1 MOI**	**2 h 1:10 MOI**	**4 h 1:1 MOI**	**4 h 1:10 MOI**
XM_034567	CCND2	cyclin D2	0.1833	0.3083	0.5300	0.2267
NM_033379	CDC2	cell division cycle 2, G1 to S and G2 to M	0.0250	0.1783	0.4483	0.5400
NM_002357	MAD	MAX dimerization protein 1	0.0150	-0.0433	-0.8050	-0.7250
XM_037657	MYC	v-myc myelocytomatosis viral oncogene homolog (avian)	0.1233	0.1783	0.6903	0.5983
NM_004725	BUB3	BUB3 budding uninhib. by benzimidazoles 3 homolog (yeast)	-0.0500	0.1467	0.4910	0.5767
XM_034725	NRP1	neuropilin 1	-0.0317	0.1583	0.2967	0.5467
XM_008679	TP53	tumor protein p53	0.6183	0.2167	0.6810	-0.1967
NM_005620	S100A11	S100 calcium binding protein A11 (calgizzarin)	0.1667	0.2750	0.4893	0.2567
NM_004705	PRKRIR	protein-kinase, interferon-inducible double stranded RNA dependent inhibitor, (P58 repressor)	0.0033	-0.0567	0.0303	0.0817

*All adjusted P-values < 0.05 Ratios represent comparisons of expression levels of non-toxigenic vs. toxigenic-challenge.

**Table 3:** Log ratios of genes of interest in ANOVA analysis* for stress-response genes

**Accession**	**Name**	**Description**	**2 h 1:1 MOI**	**2 h 1:10 MOI**	**4 h 1:1 MOI**	**4 h 1:10 MOI**
NM_004040	ARHB	ras homolog gene family, B	-0.1783	0.0233	-1.7523	-1.5083
NM_002089	CXCL2	chemokine (C-X-C motif) ligand 2	-0.2850	-0.9700	-2.3083	-1.7517
NM_001416	EIF4A1	eukaryotic translation initiation factor 4A, isoform 1	0.1067	0.3233	0.5113	0.5733
NM_005239	ETS2	v-ets erythroblastosis virus E26 oncogene homolog 2 (avian)	-0.0417	-0.1317	-0.7237	-0.6167
NM_006597	HSPA8	heat shock 70 kDa protein 8	-0.3167	-0.3550	-0.5897	-0.2633
XM_058230	JUND	jun D proto-oncogene	-0.0883	-0.0133	-0.9497	-0.7983
NM_005566	LDHA	lactate dehydrogenase A	-0.0150	0.6150	0.8707	1.1967
XM_031818	LYN	v-yes-1 Yamaguchi sarcoma viral related oncogene homolog	0.0417	-0.2733	-0.6830	-1.0617
XM_008855	NR2F6	nuclear receptor subfamily 2, group F, member 6	0.0183	0.1483	0.3897	0.4650
XM_007258	TNFAIP2	tumor necrosis factor, alpha-induced protein 2	-0.0383	-0.2517	-1.0440	-1.5067
XM_002762	TNFAIP6	tumor necrosis factor, alpha-induced protein 6	-0.0350	-0.1517	-1.5390	-1.0600
NM_003295	TPT1	tumor protein, translationally-controlled 1	-0.4950	-0.5100	-0.3893	-0.4267

* All adjusted P-values < 0.05. Ratios represent comparisons of expression levels of non-toxigenic vs. toxigenic-challenge.

**Figure 5:**
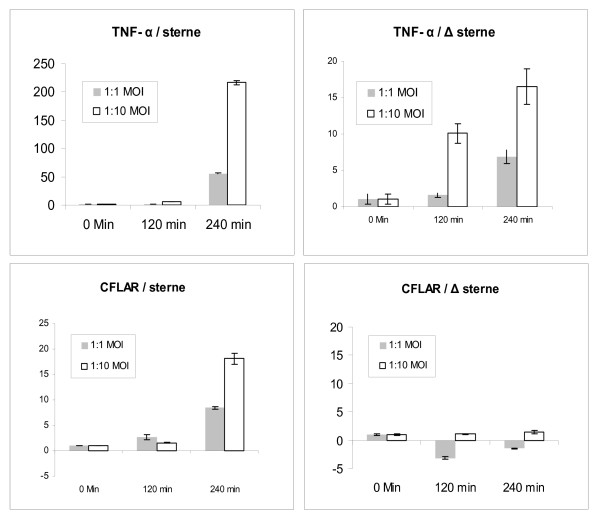
**Fold change of TNF-α and cFLAR expression in THP-1 cells as measured by Real-Time PCR.** Left panels, *B. anthracis *Sterne infection. Right panels, *B. anthracis *Δ Sterne infection. Notice the difference in the Y scale between TNF-α panels.

**Figure 6:**
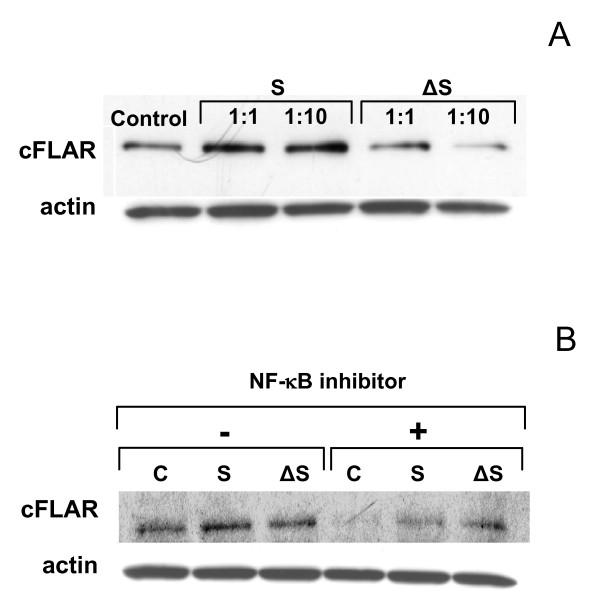
**CFLAR protein induction in THP-1 cells.** A. Western blots of CFLAR induction at MOIs 1:1 and 1:10 at 4 h post infection. B. Inhibition of NF-κB with the inhibitor QNZ during challenge suppresses the production of cFLAR. Samples were taken at 8 h post infection at MOI 1:1. C denotes unchallenged control; S, challenge with Sterne strain; and ΔS, challenge with Δ Sterne strain. Actin is shown as a control.

The ANOVA analysis shows a marked upregulation for several stress and inflammatory response genes relevant to the TNF-α biological module in the case of toxigenic Sterne spore challenge relative to the non-toxigenic Δ Sterne one (Table 1, 3). Contrasted against the apoptosis-related genes are the cell cycle genes, which seem to be affected in response to the non-toxigenic challenge (Table 2), indicating an initial shift towards proliferation and differentiation. CXCL2, the chemokine precursor to macrophage inflammatory protein-2α (MIP-2α) is strongly increased in Sterne spore-challenged cells, pointing to a higher innate response to the toxigenic strain. The apoptosis inhibitor genes for cFLAR (CASP8/FADD-Like Apoptosis Regulator) and GADD45b (Growth Arrest and DNA-Damage-inducible, beta) also appear to be expressed higher in the toxigenic spore treatments (Table 1 and Fig. 5). The level of cFLAR protein determined by Western blot follows the same trend (Fig. 6a). The increased anti-apoptotic responses to the toxigenic strain challenge correlate with the induction of higher transcription of *Nf-κB1 *(Table 1), a gene which may have some anti-apoptotic effects[31,32]. In addition the Nf-κB inhibitor QNZ reduces *cFLAR *expression (Fig. 6b) in agreement with its higher level in the toxigenic strain infection (Fig. 6a).

### TNF-α-dependent apoptosis during toxigenic infection may be countered by cFLAR-and NF-kB-dependent anti-apoptotic responses

**Figure 7:**
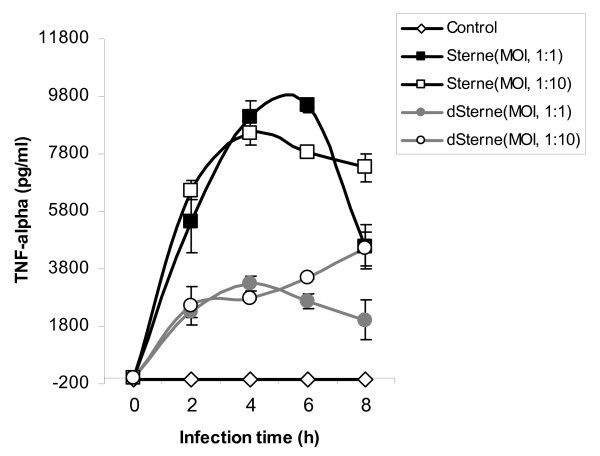
**Production of TNF-α protein mirrors transcriptional results. **ELISA of TNF-α produced by THP-1 cells challenged by Sterne and Δ Sterne over 8 h.

The role of TNF-α, the primary mediator of the cellular inflammatory response, has attracted significant attention in anthrax studies. Recent data indicate that its systemic levels during infection, or upon LeTx challenge in animals and cell cultures, are either undetectable or transient [12,20,33] and therefore cannot be considered as a primary cause of death, as it was previously suggested [34,35]. Nevertheless, some data suggest that the TNF-α response may play an important function in the local host antibacterial defense [36,37]. In our gene chip and Real-Time PCR experiments, TNF-α gene expression is significantly induced relative to uninfected cells in a time- and concentration-dependent manner during infection by both strains (Fig. 5), however the induction by the toxigenic strain is approximately 10 times higher, compared to the non-toxigenic one. This induction correlates with the increased protein levels of TNF-α determined by ELISA in the culture medium of cells after spore challenge (Fig. 7). The level of TNF-α is almost 3-fold higher (p < 0.01) in the cells challenged with the toxigenic spores after 4 h.

This large cellular TNF-α response to toxigenic spores compared to a lower one in the nontoxigenic spore-challenge is an interesting observation, suggesting that the issue of cytokine suppression during anthrax is not straightforward. It is well known that pXO1 modulates the expression of many chromosomal proteins in addition to its own, resulting in larger numbers of unique secretome proteins in pXO1 (+) strains compared to pXO1(-) strains[38]. This may contribute to the increased response to the plasmid (+) strain seen in our study. However LeTx, a well-known protease of the mitogen-activated protein kinase kinases (MEKs), would be expected to inhibit expression of cytokines, and in THP-1 cells, it has been shown to inhibit the release of TNF-α following induction by an agonist[39]. In our experiments, the expression of toxins takes place approximately 2 to 4 hours post-spore challenge, allowing time for a general innate response before cytokine suppression. Our study also agrees with a previous report from Pickering et. al., who found no inhibition of cytokine response by a toxigenic strain, and an even lower response from a non-toxigenic strain [12], while other studies, such as those by Tournier et. al., and Drysdale et. al. have demonstrated the more expected suppression of TNF-α and cytokine responses upon toxigenic *B. anthracis *challenge [40,41]. These discrepancies may in part be explained by differences in the timing mentioned above, but also by the experimental conditions. Each of these studies attempts to remove extracellular bacteria and/or excreted proteases/factors from their models in some way, therefore excluding their effect. In our conditions, we were interested in leaving these factors to allow for the evaluation of these extracellular components in the host cell response of this LeTx-cytolysis-resistant cell line.

**Figure 8:**
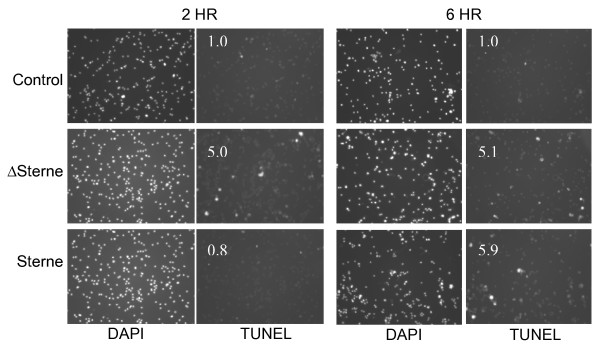
**Apoptosis of THP-1 cells is delayed at 2 h post challenge by the toxigenic Sterne strain relative to the non-toxigenic Δ Sterne strain.** Apoptosis was detected with TUNEL staining. The numbers on the images show counts of objects with the intensities above background relative to corresponding untreated controls and indicate the extent of apoptosis. DAPI staining shows that equal number of cells was used in all experiments.

Elevated TNF-α inflammatory response of the THP-1 cells to the pXO1 gene products is expected to enhance the protective, bactericidal capacity of macrophages and sensitize them to apoptosis [42,43]. It is therefore possible that *B. anthracis *has developed a mechanism to counter these macrophage defenses during the initial infectious activity. In sensitive macrophages TNF-α is expected to substantially contribute to the apoptotic death program through a TNF Receptor 1 (TNFR1)-dependent pathway including activation of caspase-8. An interesting feature of the TNF signaling network is the existence of extensive cross talk between the apoptosis and NF-κB signaling pathways that emanate from TNFR1. While in the absence of NF-κB activity the cellular susceptibility to TNF-α – induced apoptosis increases, activation of NF-κB protects against apoptosis[44,45]. Therefore, the increased TNF-α and NF-κB expression induced by the toxigenic strain may result either in the anti-apoptotic effect or in the delay of apoptosis onset depending on the balance between the above stimuli in the TNF-related apoptotic pathway. In agreement with this hypothesis, our data show that apoptosis detected by TUNEL assay is delayed after 2 h post exposure to the toxigenic strain while after 6 h it takes place to a similar extent in the case of both strains (Fig. 8).

## Discussion

Our results indicate that the THP-1 monocytes present significant innate immune responses evident in activation of several biological modules during both toxigenic, and non-toxigenic challenge. In general, the early response is suppressed by the toxigenic strain but overall this strain demonstrates stronger activation for several stress, inflammatory and apoptosis-related genes relevant to the TNF-α biological module. Observed genotypic responses specific for the pXO1-encoded pathogenic factors are consistent with mitochondrial damage, prostaglandin level disruption, and phosphatase induction likely inhibiting phosphoprotein cell signaling. These results agree with the transcriptional analysis of the murine macrophage RAW 264.7 cells in response to *B. anthracis *Sterne spore challenge [46]. The authors reported transcriptional activation of immune modules, induction of Nf-κB, along with the apoptotic inhibitor GADD45b. However in the case of un-stimulated macrophages treated with LeTx there were no significant changes observed. Analyzing the effect of LeTx on *Salmonella *lipopolysaccharide-stimulated RAW 264.7 cells, Tucker *et al*. detected suppression of the host immune response [47] however the antigen (LPS) used for stimulation is irrelevant to anthrax infection. According to our results, the plasmid-negative strain provides the background information, which is important for precise evaluation of the pXO1 contribution to the host cell response, such as silencing of protective signaling in response to infection and/or changes in signaling mechanisms.

We present evidence that both toxigenic and non-toxigenic *B. anthracis *strains induce apoptosis, which can be considered as a form of innate immunity against some pathogens. Our data indicate that in the absence of toxigenic pXO1, the infection with the avirulent strain proceeds without a substantial intrinsic insult to the cell. Therefore in the absence of cFLAR induction, and in conjunction with the observed TNF-α production, we hypothesize that infection in the absence of pXO1 probably induces apoptosis through a classical extrinsic TNF-α receptor-mediated caspase-8 pathway. In contrast, the pXO1-encoded complex of secreted toxins acquired by *B. anthracis *as an instrument of intracellular immune evasion causes a strong intracellular insult. The large induction of CFLAR likely plays some role in inhibiting TNFR-mediated apoptosis, and may be involved in its observed phenotypical delay, ultimately pushing the system toward the intrinsic, mitochondria-dependent pathway of apoptosis some time later [48]. This pathway proceeds through mitochondrial dysfunction, and is a primary differential observation of our gene ontology modules between the strains. (Fig. 4), as indicated by the perturbation of modules containing mitochondrial components in the Sterne-infected cells. Future experimentation such as the TNFR1-dependent processing of caspase-8 *vs*. caspase-9, mitochondrial membrane polarization/permeability, and the formation of apoptotic signaling complexes during challenge by each of these strains will help to determine if this is indeed the case.

Interestingly, the onset of apoptosis induced by a toxigenic strain is markedly delayed, and bacterial growth is significantly increased, indicating existence of a mechanism modulating apoptosis in favor of the microbe through the increased production of anti-apoptotic cFLAR and Nf-κB. This delay of apoptosis during anthrax infection could allow for more successful bacterial survival and dissemination, resulting in systemic disease. This supports a hypothesis that in anthrax the suppression of apoptosis in macrophage-like cells may contribute to disease virulence during the initial stages of infection, and that apoptosis itself may represent a protective host cell response until it reaches a pathologic proportion.

Several lines of evidence in the literature support this hypothesis. Previous analyses of anthrax-infected RAW 264.7 cells [46] identified overexpression of ornithine decarboxylase (ODC), a normal biosynthetic enzyme involved in the conversion of putrescine to the polyamines spermine and spermidine. Since the overproduction of polyamines by ODC has been implicated in preventing apoptosis [49], this led the authors to speculate that ODC overexpression was involved in suppression of macrophage apoptosis. In the case of other bacterial infections, apoptosis has been generally considered as a protective host response [50]. There is additional evidence that the delay of apoptosis may play roles in some viral and fungal infections as well [51-53].

Macrophages infected with viable virulent strains of *Mycobacterium tuberculosis *undergo apoptosis with far less frequency than macrophages infected with attenuated strains [21,22]. Strain virulence in mice correlates directly to the extent to which apoptosis is inhibited [23]. Thus virulent strains of *M. tuberculosis *apparently suppress apoptosis in host cells. Conversely, macrophage apoptosis appears to provide the host with a means for killing intracellular microorganisms, with host cells likely initiating apoptosis following initial infection [54,55]. Consistent with this, *Mycobacterium Avium*, a less virulent pathogen that is less adapted to the intra-macrophage growth species compared to *M. tuberculosis *does not suppress macrophage apoptosis [56].

Mycobacterium infections have been indicated to subvert pro-apoptotic TNF-α signaling by either reduction of *TNF-α *expression or downregulation of its receptor [57], as well as differential NF-κB activation [58]. It was found that both virulent and avirulent *M. tuberculosis *strains activated NF-κB after 4 h in THP-1 cells however after 48 h only the virulent strain maintained NF-κB activation, which lead to up-regulation of a bcl-2 family anti-apoptotic member, bfl-1/A1. These results indicate that NF-κB activation may be a determinant factor for the success of virulent mycobacteria within macrophages. *L. pneumophila *induces a robust activation of caspase-3 in alveolar macrophages however the apoptotic cell death is not executed until late stages of the infection, concomitant with the termination of intracellular replication [59].

Recent studies of hepatitis C infection directly implicate the sustained upregulation of the *cFLAR *gene product in preventing TNF-α-mediated apoptosis and contributing to disease initiation by the virus core protein [52]. The induction of cFLAR during toxigenic anthrax infection likely contributes to prevent apoptosis in a similar fashion, particularly when combined with the suppression of TNF-α production, as it has been shown that LeTx inhibited the TNF-α production in both endotoxin-stimulated macrophages [60,61] and anthrax bacterial cell wall-stimulated peripheral blood mononuclear cells [6]. A mechanism consistent with our observations has been suggested for THP-1 cells stimulated through TLR-4 with the pore-forming hemolysin, anthrolysin O (AnlO) [54]. In this report the NF-κB-dependent activation of *Bfl-1/A1 *gene by AnlO contributed to anti-apoptotic signaling, although overall the TLR-4 stimulation resulted in apoptosis. However the role of AnlO *in vivo *remains to be elucidated. Another possible pathway of apoptosis inhibition in macrophages includes EdTx-mediated activation of the anti-apoptotic Platelet Activator Inhibitor type 2 gene (*PAI-2*), although THP-1 cells carry an inactivating mutation in this gene [46].

The previous findings that anthrax LeTx inhibits the p38 signaling pathway by cleavage of MAPKKs in macrophages and dendritic cells [60,62] are consistent with the protective role of this stress-activated pathway in elimination of intracellular pathogens and activation of host immunity [63]. From this standpoint, the proteolytic activity of LeTx on the MAPK activation pathway may contribute to establishing infection by preventing cell activation in response to infection and thereby increasing the bacterial survival within phagocytes [19,60].

## Conclusion

Using a matched pair of the pXO1 (+) and pXO1 (-) *B. anthracis *strains, we demonstrate that the monocyte-type THP cells *in vitro *generate a distinct set of transcriptional responses to infection characteristic of the virulence factors contributed by the XO1 plasmid. Among these responses, the delay of apoptosis associated with the elevated expression of anti-apoptotic cFLAR represents a likely pathogenic strategy directed toward bacterial survival in the conditions of the increased host inflammatory response. This may be particularly relevant for hosts carrying mononuclear cells which, like THP-1, are resistant to LeTx-induced cytolysis. In the absence of pXO1, the non-toxigenic strain induces early apoptosis consistent with its protective role in several infectious diseases as a form of the host innate immune response. This finding implies that in addition to LeTx, virulent *B. anthracis *possesses a chromosome-encoded factor(s) triggering a pro-apoptotic host response, as well as the plasmid factors counterbalancing apoptosis.

## Methods

### Bacterial strains and THP-1 cell cultures

*Bacillus anthracis *strain 34F2 Sterne (pXO1^+^, pXO2^-^) was obtained from Colorado Serum Company (CO). Δ Sterne strain, from the Collection of the National Center for Biodefense and Infectious Diseases (George Mason University, Manassas, VA), is a plasmidless derivative of Sterne strain generated by curing of the pXO1 plasmid by growth for 6 days in LB media at 42.5°C [64]. In our experiments, the absence of pXO1 in the cured strain was confirmed by PCR with the protective antigen-specific primers. *B. anthracis *is one of the most monophyletic bacterial strains known, however the introduction of genomic changes by the curing process cannot be discounted. Despite the uniformity of its genome, *B. anthracis *strains do contain sequences which exhibit variability in response to selective pressures and allow differentiation. As it is not possible to test all genomic sequence without considerable time and expense, we therefore applied a high-resolution multi locus sequence typing using the technique and primers of Helgeson [65] to ensure that both strains were at least identical for all variable sequences tested (Additional file 2) Growth curves for both strains were also identical in 1% fetal bovine serum (FBS) cell culture medium, with 4 h corresponding to the mid point of the exponential phase of growth (Additional file). Spores were generated by growth for 4 days on LB agar plates followed by re-suspension in water and pelleting by centrifugation two to four times, then storage in water at 4°C until use.

THP-1 was obtained from ATCC (Manassas, VA) and grown in complete medium (RPMI 1640 with glutamine, 25 mM HEPES, 4.5 g/l glucose, and 10% FBS) for propagation, until 24 h before challenge with spores, at which point THP-1 cells were washed and re-suspended for 24 h in serum-free (low protein) Cellgro media (Media Tech, Herndon, Va.) at a concentration of 1.0 × 10^6 ^cells/ml. Cell cultures were challenged for 2 h or 4 h at a cell to spore multiplicity of infection (MOI) of either 1:1 or 1:10 for microarray experiments. The term MOI used here simply refers to the number of spores added per cell. Each timepoint/MOI combination was replicated at least 3 times for OD readings and microarray analysis. Viability was measured by a standard Trypan Blue permeability assay by counting dead cells with a hemocytometer. Infection of THP-1 monocytes by both strains for 4 h in the conditions of our experiments resulted in a viability of greater than 90% relative to untreated cells.

### Microarrays and ANOVA analysis

RNA was collected and purfied immediately at the appopriate timepoints by Trizol (Invitrogen Corporation, Carlsbad, CA), and quality determined by checking on a Lab-on-a-Chip Bioanalyzer 2100 (Agilent Technologies, Santa Clara, CA). Samples were similarly quality checked after both probe labelling and hybridizations steps. The microarray platform was developed, printed, hybridized and analyzed at Avalon Pharmaceuticals, Inc.(Germantown, MD). Probe design and printing were all done using automated, proprietary processes (Avalon Pharmacueticals, Germaintown, Md) and contained quality control steps after each. Arrays consist of proprietary 80-mer long, spotted oligonucleotides representing 2,000 human genes. Each chip contains four replicates of each gene (8,000 total/chip) with 64 perfect match and 64 mismatch controls. Arrays were hybridized using standard Cy3/Cy5 sample/reference labeling and analyzed using a ScanArray reader (Perkin-Elmer, Wellesly, MA). Several sets of genes related to apoptosis, stress response, and cell differentiation were chosen for further evaluation. To determine whether these genes were differentially expressed between two experimental states, an ANOVA was performed on the log-transformed expression ratios as described in [66]. The following model was used for this analysis: *y*_*ijkl *_= *E*_*i *_+ *M*_*j *_+ *T*_*k *_+ (*EMT*)_*ijkl *_+ *ε*_*ijkl *_where *y*_*ijkl *_denotes the log_2 _expression ratio measured for experimental state *i*, MOI *j*, time state *k*, replicate *l*, with *1 *≤ *i *≤ *3*, *1 *≤ *j *≤ *2*, *1 *≤ *k *≤ *2*, and *1 *≤ *l *≤ *6*. The term *E*_*i *_measures the effect of the infection; *M*_*j *_measures the effect of the MOI; *T*_*k *_measures the effect of the time state, and the term (*EMT*)_*ijkl *_measures the interaction effect of all three variables. An ANOVA was performed on each gene using the linear model above. Twelve contrasts were based on pairwise differences between each experimental condition and the control state, as well as the experimental state which differed in the MOIs only. The R package limma was used for ANOVA methods [67]. A multiple testing correction [68] controlled the false discovery rate, which is the expected proportion of false positives in the rejected hypotheses. Genes with adjusted F-statistic *p*-values < 0.05 were collected for further inspection.

### Real-Time PCR

To confirm microarray data, Real-Time PCR was performed using the iQ SYBR Green kit from BIO-RAD (Hercules, CA) using the manufacturers specifications. Real time data for each gene represents the average of 3 biological replicates, each replicated 3 times for a total of 9 reactions per gene/treatment. Fold expression was calculated for each gene relative to an endogenous 18-S control gene using the 2^-ΔΔCT ^method as described[69]. Primers chosen for Real-Time analysis were as follows: The human 18-S ribosomal gene (*18S*), 5'AGGAATTCCCAGTAAGTGCG and 5'GCCTCACTAAACCATCCAA; Tumor necrosis factor-α (TNF superfamily, member 2; *TNF-α*), 5'TGTCTCAACCCCGCATCG and 5'AGGAACAGCCACCAATAAGC; Ras-related C3 botulinum toxin substrate 2 (Rho family, small GTP-binding protein Rac2; *RAC2*), 5'CCTCATCATCCAGTCCAACG and 5'GGCGAACTCCTGCTCCTC; Chemokine (C-X-C motif) ligand 2 (CXCL2), 5' TGTCTCAACCCCGCATCG and 5' AGGAACAGCCACCAATAAGC; CASP8/FADD-like apoptosis regulator (*cFLAR*), 5'AGATCACCAGCCTTACCG and 5'GATGAGCCACAGGAAATGC. T_m_s used in Real-Time cyclers were 54.3°C for *TNF-α*, *cFLAR*, and *18-S *genes; 52.9°C for the *RAC *gene; and 63.2°C for the CXCL2 gene.

### Representation of biological response modules

Expression values of genes from each of three replicate chips for each treatment were averaged, and genes corresponding to specific gene ontology (GO) categorizations (modules) . were clustered using '*Cluster*' [34,70]. Clustering was performed without additional filtering or adjustment, using the uncentered correlation coefficient, followed by complete linkage clustering.

### Western Blot Analysis and ELISA

For Western blot analysis, spore-treated THP-1 cells were lysed in a buffer containing 25 mM Tris-HCl, pH 7.6, 150 mM NaCl, 1% NP-40, 1% sodium deoxycholate and 0.1% SDS (RIPA buffer, Pierce) and cell debris was removed by centrifugation. Resulting proteins were separated in a 4–20% gradient gel and electrophoretically transferred to a PVDF membrane. The membrane was probed with mouse monoclonal antibodies against cFLAR (Abnova), and actin (Chemicon), and then the horseradish peroxidisase-conjugated anti-mouse secondary antibody. To quantify TNF-α proteins secreted into culture medium, human TNF-α ELISA was performed according to the manufacturer's recommendation (BD Biosciences). The culture supernatants were collected by centrifugation of THP-1 cells cultured in a serum-free medium at the indicated time point post spore challenge. To investigate whether NF-kB is involved in cFLAR expression, 10 μM NF-kB inhibitor 6-amino-4-(4-phenoxyphenylethylamino) quinazoline, a cell-permeable quinazoline (QNZ, Sigma), was added to THP-1 cell culture 30 min prior to treatment with spores (MOI 1). After incubation for 8 h, cells were lysed in RIPA buffer and analyzed by Western blotting as described above.

### Apoptosis Detection

For detection of apoptosis, terminal dUTP nick-end labeling (TUNEL) assay was performed according to manufacturer's recommendation (Roche Applied Science). Briefly, the spore-challenged THP-1 cells were washed 3 times with phosphate-buffered saline (PBS), and fixed with 2% paraformaldehyde for 60 min at room temperature. The cells were washed with PBS and permeabilized with 0.1% Triton X-100 in 0.1% sodium citrate for 2 min. TUNEL reaction was carried out for 60 min at 37°C in a humidified atmosphere by adding 50 μL of reaction mixture containing terminal deoxynucleotidyl transferase. The TUNEL-positive cells were analyzed under a fluorescence microscope. The number of objects with the intensities above background on the images was determined using the Elements software (NikonNicon).

## Authors' contributions

CB conceived this study, drafted the manuscript, generated the pXO1(-) strain, and performed the bacterial challenges of cell culture, qPCR, and GO clustering. MCC performed the inhibitor experiments/Western Blots, ELISAs, and TUNEL assays. QZ and DL performed all microarray experiments and generated PCA, Variance, gene lists and GO profiles of results. KS performed ANOVA and contributed portions of the manuscript. TP grew and prepared anthrax strains and performed bacterial challenge experiments. AP performed qPCR experiments. CB and DS provided material support and encouragement for this work. SP provided material support and direction, and drafted significant portions of the manuscript.

## Supplementary Material

Additional file 1 **Growth curves of *B. anthracis *Sterne and *B. anthracis *Δ Sterne in the conditions of THP-1 cells cultivation in serum-free medium supplemented with 1% FBS at 37°C. **The data represent a comparison of growth curves for pXO1 (+), and pXO1 (-) strains. Click here for file

Additional file 2 **Results of multi locus sequence typing (MLST) by method of**[58]. The data represent a comparison of sequence homology between pXO1 (+), and pXO1 (-) strains, at certain variable sequence regions on the chromosome. Click here for file

Additional file 3 **Log ratios of genes evaluated in ANOVA (all adjusted P-values < 0.05).** The data represent a comprehensive list of genes evaluated in ANOVA. Click here for file
