# Supplementary material for: Transcriptional and apoptotic responses of THP-1 cells to challenge with toxigenic, and non-toxigenic Bacillus anthracis
Source: BMC Immunol. 2008 Nov 13;9:67. doi: 10.1186/1471-2172-9-67 (PMC2613145; doi:10.1186/1471-2172-9-67)
Supplement: Additional file 2 — Results of multi locus sequence typing (MLST) by method of[58]. The data represent a comparison of sequence homology between pXO1 (+), and pXO1 (-) strains, at certain variable sequence regions on the chromosome. [file 1471-2172-9-67-S2.doc]

Table A1. Results of multi locus sequence typing (MLST) by method of [58].*

| MLST and pXO1 genes | *B. anthracis* Sterne | *B. anthracis* Delta Sterne | ST homology (%) |
| --- | --- | --- | --- |
| adk | + | + | 100 |
| ccpA | + | + | 100 |
| ftsA | + | + | 100 |
| glpT | + | + | 100 |
| pyrE | + | + | 100 |
| recF | + | + | 100 |
| sucC | + | + | 100 |
| PA | + | - | n/a |

* The primers used correspond to the 330- to 504-bp conserved internal regions of the housekeeping genes. The cured and the parental strains are identical for all variable sequences tested except PA, which is not present in Δ Sterne.
