# Supplementary material for: Transcriptional and apoptotic responses of THP-1 cells to challenge with toxigenic, and non-toxigenic Bacillus anthracis
Source: BMC Immunol. 2008 Nov 13;9:67. doi: 10.1186/1471-2172-9-67 (PMC2613145; doi:10.1186/1471-2172-9-67)
Supplement: Supplementary file 2 [file 1471-2172-9-67-S1.doc]

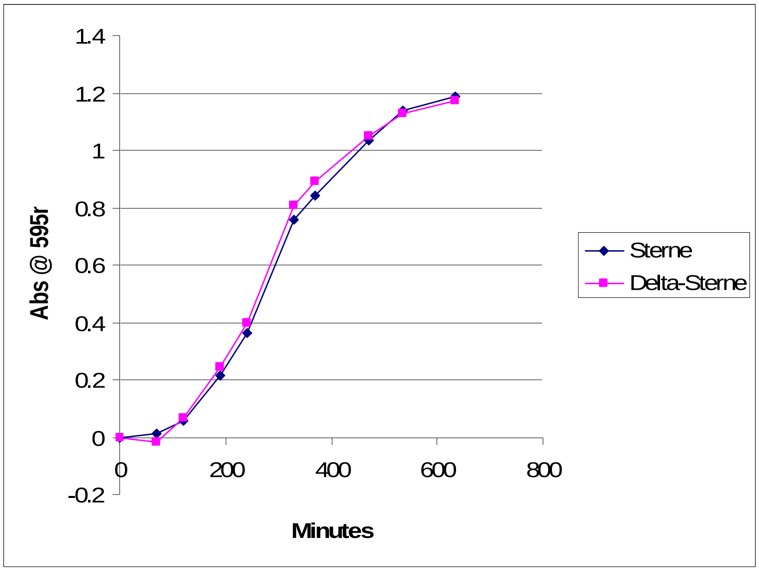

1.4
1.2
1
0.8
0.6
0.4
0.2
0
-0.2
0
200
400
600
800
Abs @ 595r
Minutes
Sterne
Delta-Sterne
